# Predictors of hospital nursing staff’s adherence to safe injection guidelines: application of the protection motivation theory in Fars province, Iran

**DOI:** 10.1186/s12912-023-01687-x

**Published:** 2024-01-09

**Authors:** Masoud Karimi, Zakieh Khoramaki, Maryam Rabiey faradonbeh, Maryam Ghaedi, Fatemeh Ashoori, Abdolrahim Asadollahi

**Affiliations:** 1 Research Center for Health Sciences, Institute of Health, Department of Health Promotion, School of Health, Shiraz University of Medical Sciences, Shiraz, Iran; 2 Department of Health Promotion, School of Health, Shiraz University of Medical Sciences, Shiraz, Iran; 3 Research Center for Health Sciences, Institute of Health, Department of Gerontology, School of Health, Shiraz University of Medical Sciences, Shiraz, Iran

**Keywords:** Needle-Stick Injuries, Motivation, Nursing staff, Guideline Adherence

## Abstract

**Background:**

Needle stick injuries (NSI) can lead to dangerous infectious diseases for health care workers. This study aimed to determine the predictors of observance of safe injection guidelines in hospital nursing staff, based on protection motivation theory.

**Methods:**

This cross-sectional study was conducted on the nursing staff of five randomly selected teaching and general hospitals of Shiraz University of Medical Sciences, Fars province, Iran, in 2021. Data were collected using a researcher-made questionnaire which was developed based on protection motivation theory. Data were processed and analyzed using SPSS 22 and Amos 24 at a significance level of  < 0.05. Pearson’s correlation coefficient, Multiple linear regression, and Structural Equation Modeling were used.

**Results:**

The mean age of the participants (No = 280) was 32.5 ± 8.09 years. Most of the participants [80%] had encountered NSI, patients’ blood, or body fluids at least once. Protection motivation was the only predictor of protective behaviors (β = 0.573), while perceived efficacy (β = 0.142) and perceived rewards (β = -0.229) were the strongest predictors of motivation. The structural equation modeling analysis showed that standardized total effects of protection motivation, perceived response costs, rewards, and efficacy on the protective behaviors were 0.573, -0.159, -.104, and 0.81, respectively. While standardized total effects of perceived rewards, efficacy, and response cost on protection motivation were -0.229, 0.142, and -0.033, respectively. The model fit indices indicated the acceptable final model fit.

**Conclusion:**

The results suggest that perceived efficacy, perceived effectiveness, and perceived rewards were the most important predictors of adherence to Safe Injection Guidelines in nursing staff.

**Supplementary Information:**

The online version contains supplementary material available at 10.1186/s12912-023-01687-x.

## Introduction

Healthcare workers are exposed to blood and other body fluids daily at work due to needle stick injuries or splashing patient discharges into their eyes, nose, or mouth [1]. These injuries may transmit dangerous infectious diseases including hepatitis B, hepatitis C, and HIV/AIDSF [2]. Needle stick injuries [NSI] which are defined as “a puncture wound, cut, of scratches inflicted by medical instruments intended for cutting or puncturing [cannula, lancets, scalpels, etc.]” account for about 70% of these cases [3].

The US Centers for Disease Control and Prevention have estimated that 385,000 injuries occur per year for hospital staff [4]. Every year, 10 percent of the hospital staff experience needle stick injuries, worldwide [1, 5]. Different studies reported prevalence of needle stick injuries between 49 to 54 percent in public and teaching hospitals in Fars province, Iran [6–8].

Adherence to standard precautions and following the relevant guidelines will reduce the risk of needle stick injuries and occupational contact with the patient's blood and other body fluids [4, 9, 10]. Several studies have considered educational intervention for nurses as one of the most important methods and strategies to reduce needle stick injuries [11–15].

The most important factor influencing needle stick prevention behaviors is the attitude of nurses, which should be changed through proper training [16]. Theories and models of human behavior change provide strong frameworks for understanding how people learn, what attitudes they have about the behavior, and how and why they engage in specific behaviors [17, 18]. One of these theories is the protection motivation theory (PMT) which was proposed by Rogers (1975), as a cognitive framework for examining and understanding the causes of health-protective behaviors and explaining the persuading mechanisms of fear appeals [19, 20].

Protection motivation theory consists of five main constructs, including perceived threats composed of a combination of individuals’ perceptions about the likelihood of getting a disease or condition (perceived susceptibility) and the seriousness of that condition’s consequences (perceived severity) [18, 21]; perceived rewards which are defined as the benefits or advantages which individuals may experience by doing risky behaviors, and may be extrinsic (social approval) or intrinsic (e.g. Pleasure or Accelerating work) [22]; perceived efficacy as a combination of individuals’ perception about the effectiveness of recommended behavior in diminishing the threat (response efficacy) and their ability to complete the behavior despite the barriers and costs (self-efficacy) [20, 23]; response costs including any costs (monetary, personal, time, effort) that hinder the individuals from doing recommended behaviors [20, 24]; and protection motivation or intention to perform the recommended behaviors [22, 24].

Multiple studies have been conducted to investigate the factors affecting compliance with safety guidelines in the provision of nursing services; however, most of them focused on demographic, cultural, and organizational factors [25–27]. In the few studies conducted to investigate the individual level factors which were effective on adherence to safe injection guidelines based on behavior change models and theories, response efficacy, self-efficacy, response costs [28, 29] significantly predicted the protective behaviors in nurses or laboratory staffs.

Thus, given the importance of needle stick injuries for healthcare workers and the community, and considering that few studies have investigated the effects of individual factors of the nursing staff (such as attitudes and beliefs) on compliance with the principles of safe injections, this study was conducted to examine the predictors of the adherence to standard guidelines for safe injections as a protective behavior using the protective motivation theory among hospital nurses. Accordingly, seven hypotheses were tested:

- H1. Protection motivation has a direct, positive and significant relationship with adherence to safe injection guidelines.
- H2. Perceived threat has a direct positive and significant relationship with adherence to safe injection guidelines.
- H3. Perceived rewards have a direct negative and significant relationship with adherence to safe injection guidelines.
- H4. Perceived efficacy has a direct, positive, and significant relationship with adherence to safe injection guidelines.
- H5. Perceived cost has a direct, negative, and significant relationship with adherence to safe injection guidelines.
- H6. Perceived fear has a direct, negative, and significant relationship with adherence to safe injection guidelines.
- H7: Experience of exposure to NSI has a direct positive and significant relationship with adherence to safe injection guidelines.

## Materials and methods

In this cross-sectional study, which was conducted in Fars province, Iran, in 2021(May to July), the study population was all the nursing staff (nurses, midwives and operating room staff) of Shiraz University of Medical Sciences. Having at least 3 months of work experience, employment in medical departments for at least 12 h per week, and consent to participate in the study were considered as inclusion criteria, and the cases who did not fill out the questionnaire were excluded from the study.

Based on the estimated prevalence of needle stick in Shiraz teaching hospitals in previous studies [6–8], and using NCSS PASS 15, a sample size of 280 was calculated for the study (α = 0.05, d = 0.05, non-response rate 10%). Participants were enrolled using multistage random sampling method. For this purpose, first, five teaching and general hospitals of Shiraz University of Medical Sciences were randomly selected from 47 hospitals; then, the participants were selected based on the number of staff in each hospital through simple random sampling method.

The data collection tool was a researcher-made questionnaire consisting of three parts: The first part is a demographic information form (age, sex, work experience, level of education, field of study, and awareness about safe injection guideline). The second part contains information about the participants’ experiences about occupational exposure to needle sticks, patients' blood and fluids during their career time, including three items (with not at all, once, twice, three times, four and more times options). The third part includes a researcher-made questionnaire which was developed based on protection motivation theory, including perceived threat (6 items), internal and external rewards (6 items), response costs (3 items), perceived efficacy (6 items), and fear (3 items) all using a 5-point Likert scale (strongly agree to strongly disagree), protection motivation (7 items) using a 5-point Likert scale (not at all to definitely), and finally preventive behaviors (7 items) with four-point scale (not at all, rarely, sometimes, always).

Seven behaviors assessed in this study were using a protective pad when breaking vials, wearing gloves, glasses, mask and plastic apron to protect the body against exposure to blood or body secretions, Not putting a needle head cover on it and avoiding breaking or bending the needle head before disposing of it.

The face validity of the questionnaire was confirmed by ten nurses who were interviewed face-to-face and examined the items in terms of the level of difficulty, appropriateness, and ambiguity. Content validity ratio (CVR) and content validity index (CVI) were calculated to evaluate the content validity of the questionnaire, using the opinions of a panel of experts consisting of six health education and promotion professionals and four nurses. The CVR of the items was rated between 0.75 and 1.0, what was acceptable based on Lawshe criteria [30], and CVI of the items rated between 0.81 and 1.0 which was acceptable based on Waltz and Bussel’s criteria [31]. Internal consistency of the questionnaire was calculated through Cronbach alpha (> 0.62) for each construct. The external consistency was assessed by intra-class correlation [ICC] in a test- retest on a pilot study with two weeks’ interval (*N* = 30, ICC = 0.71, *P* = 0.01). The value of item-total correlation coefficient of greater than 0.40 for each study construct was considered as adequate evidence of convergent validity. Discriminant validity was supported whenever a correlation between an item and its hypothesized construct was higher than that with the other constructs [32]. Table 1 shows that all the domains have success rates for convergent and discriminant validity.

**Table 1:** Convergent and discriminant validity for the designed questionnaire

Construct	No. items	n	Convergent validity (Range of correlations)	Discriminant validity (Range of correlations)
Perceived Threat	6	270	0.41–0.45	-0.005–0.28
Perceived rewards	6	270	0.57–0.76	-0.566–0.39
Perceived efficacy	6	270	0.42–0.77	-0.47–0.51
Perceived costs	3	270	0.81–0.86	-.538–0.41
Perceived fear	3	270	0.82–0.91	-0.57–0.339
Protection motivation	7	270	0.52–0.72	-0.27–0.25
Preventive behaviors	7	270	0.49–0.67	-0.012–0.239

Because of Covid-19 pandemic, questionnaires were administered online using Porsline, which is an online survey platform in Iran. For this purpose, the educational supervisor of each hospital was asked to send the online questionnaire to the selected personnel, and each personnel could answer the questionnaire only once on his/her mobile phone.

Data were analyzed using SPSS 22 statistical software at a significance level of  < 0.05. The normality assumption of the variables was checked and confirmed through Kolmogorov-Simrnov test. Frequency descriptive statistics were used to report the frequency of the participants' demographic information. The correlation between the mean scores of constructs were analyzed and reported by Pearson’s correlation coefficient. Multiple linear regression analysis was used to identify the factors predicting protection motivation and NSI preventive behaviors. Structural Equation Modeling (SEM) by AMOS 24 software was used to evaluate the fit indices of proposed model. Goodness-of-fit indices such as Chi-Square/Degrees of Freedom Ratio (X^2^/Df), Root Mean Square Error of Approximation (RMSEA), Goodness-of-Fit Index (GFI), Comparative Fit Index (CFI), and Incremental Fit Index (IFI) were used to assess the final model fit [33, 34].

## Results

Among 280 distributed questionnaires, 270 were responded completely and entered in data analysis (response rate = 96.4%). The mean age of the participants was 32.5 ± 8.09 years. While the experience of exposure to NSI, blood, and body fluids at least once were reported 56(24.2%), 36(13.4%), and 41(15.2%) participants, respectively. 56(20.7%)participants had reported no experience of NSI or exposure to patients’ blood or body fluids during their career time. The frequency distributions of the participants’ demographic characteristics are presented in Table 2.

**Table 2:** Frequency distributions of the participants’ demographic characteristics

Variable		N	Percent
Number of participants in each hospital	Hafez	60	22.2
	Ali Asghar	16	5.9
	Shahid Dastgheib	39	14.4
	Valie-Asr (Eqlid city)	25	9.3
	Shahid Faghihi	130	48.2
Sex	Male	86	31.9
	Female	184	68.1
Profession	Nurse	222	82.2
	Others (midwife, operating room, …)	48	17.8
Education level	Student	9	3.4
	Associate degree	12	4.5
	Bachelor degree	233	87.3
	Master degree	13	4.9
Work experience	< 2 years	79	31.0
	2–5 years	46	18.0
	5–10 years	45	17.6
	10–15 years	22	8.6
	> 15 years	63	24.7

Bivariate Pearson correlation analysis showed that there was a moderate positive significant correlation between protection motivation and behaviors (*r *= 0.558, *P* < 0.001), but perceived efficacy (*r* = 0.180, *P* = 0.003) and response cost (*r* = -0.155, *P* = 0.012) had a weak, positive, and negative significant correlation with protective behaviors. On the other hand, perceived rewards (*r *= -0.214, *p* < 0.001) and response costs (*r* = -0.213, *P* < 0.001) had a weak and negative correlations with protection motivation (Table 3).

**Table 3:** Matrix of correlation coefficient between protection motivation theory constructs and NSI preventing behaviors

Variables	Mean (SD)	1	2	3	4	5	6	7
1. Behavior	20.92(8.09)	1						
2. Protection Motivation	30.64(5.39)	0.558^**^	1					
3. Perceived threat	23.55(3.11)	0.026	0.094	1				
4. Perceived rewards	15.86(5.24)	-0.077	-0.214^**^	-0.108	1			
5. Perceived efficacy	22.96(4.93)	0.180^**^	0.166	0.310^**^	-0.198^**^	1		
6. Response costs	8.32(3.19)	-0.155^*^	-0.213^**^	-0.095	0.726^**^	-0.225^**^	1	
7. Fear	11.76(2.43)	0.060	0.014	0.311^**^	0.149^*^	0.148^*^	0.128^**^	1
8. Exposure	4.95(3.90)	0.050	0.079	0.040	-0.012	-0.012	0.082	0.201^**^

Multiple linear regression analysis was done in two steps; in the first step (Table 4) the protective behavior was assumed as dependent variable, and the results revealed that protection motivation was the only significant predictor of protective behavior (β = 0.50, *p* < 0.001); however, in the second step in which protection motivation was assumed as the dependent variable (Table 5), perceived rewards (β = -0.205, *p* = 0.027) and perceived efficacy (β = 0.148, *p* = 0.025) were negative and positive significant predictors of protection motivation, respectively. It is worth mentioning here that the model accounted for 29% of the variance in the protective behaviors and 7% of the variance in protection motivation.

**Table 4:** Multiple linear regression analysis of PMT constructs affecting NSI preventing behaviors

Variables	Unstandardized coefficients		Standard B	t	sig
	B	SE			
Constant	8.111	2.445	-	3.318	0.001
Protection Motivation	0.415	0.046	0.500	9.034	< 0.001
Perceived threat	-0.127	0.078	-0.096	-1.632	0.104
Perceived rewards	0.109	0.063	0.140	1.723	0.086
Perceived efficacy	0.088	0.047	0.109	1.864	0.063
Response costs	-0.175	0.101	-0.137	-1.724	0.086
Fear	0.080	0.097	0.048	0.822	0.412
Exposure	-0.206	0.058	-0.025	-0.440	0.660

Adjusted R^2^ = 0.288 *p* < 0.001

**Table 5:** Multiple linear regression analysis of PMT constructs affecting NSI protection motivation

Variables	Unstandardized coefficients		Standard B	t	sig
	B	SE			
Constant	30.158	2.796	-	10.786	< 0.001
Perceived threat	-0.030	0.197	-0.019	-0.280	0.780
Perceived rewards	-0.191	0.085	-0.205	-2.223	0.027
Perceived efficacy	0.146	0.065	0.148	2.249	0.025
Response costs	0.004	0.140	0.003	0.030	0.976
Fear	0.060	0.135	0.030	0.448	0.654
Exposure	0.082	0.080	0.065	1.022	0.308

Adjusted R^2^ = 0.07 *p* = 0.005

**Fig. 1:**
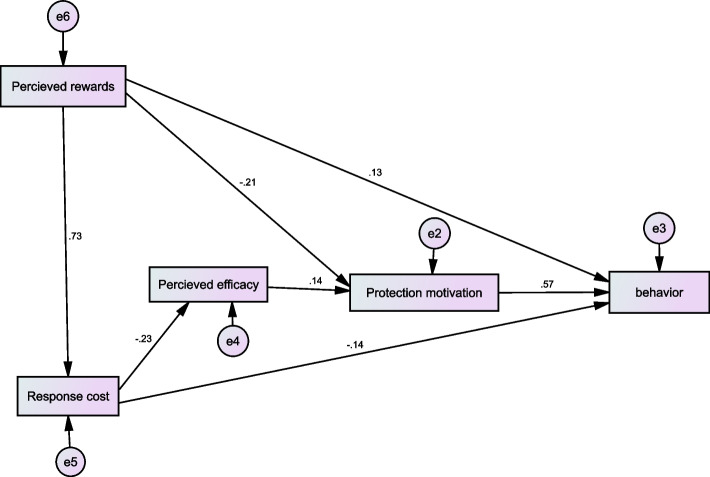
Standardized parameter estimates for the final model

The findings of structural equation modeling analysis showed that standardized total effects of protection motivation, perceived response costs, rewards, and efficacy on the protective behaviors were 0.573, -0.159, -0.104, and 0.81, respectively. While standardized total effects of perceived rewards, efficacy and response cost on protection motivation were -0.229, 0.142, and -0.033, respectively. Perceived threat and exposure experiences did not enter the model (Fig. 1). The model fit indices indicated the acceptable final model fit (x^2^ / df = 1.276, *p* = 0.281, NFI = 0.989, CFI = 0.997, IFI = 0.0.998, PCFI = 0.199, PNFI = 0.198, RMSEA = 0.032).

## Discussion

This study aimed to determine the predictors of adherence to standard guidelines for safe injections as a protective behavior, using protection motivation theory in 2021. The findings showed that at least 56% of the participants had NSI experience during their working years, while in the study of Askarian et al. (2007), in which 1555 nurses from Fars Province, Iran, participated, the NSI prevalence was reported to be 52.6% [7]. The prevalence of NSIs in the total work experience and the last year in the study of Jahangiri et al. (2016) was 76% and 54%, respectively [8].

Findings of the present study showed that there was a moderate positive and significant correlation between the participants’ protection motivation and their preventive behaviors; this finding was consistent with those of the studies of Ismara et al. (2019) in Indonesia [35] and Hosseini et al. (2017) in Iran [29]. Multiple behavioral change theories such as Theory of Reasoned Action (TRA) and the Theory of Planned Behavior (TPB) also indicate that motivation is the main determinant of the likelihood of performing specific behaviors [18].

In the present study, perceived rewards had a weak negative correlation with protection motivation. In the study of Bashirian et al. (2020) [36], 761 healthcare workers were asked about the reasons for observing COVID-19 preventive behaviors; there were positive and significant correlations between the intention of preventive behaviors and other constructs of PMT. This is not consistent with the findings of the present study. This might be due to differences in studied behaviors. On the other hand, consistent with our findings, Hosseini et al. (2017) [29] and Ezati Rad et al. (2021) [37] reported negative correlations between perceived rewards and protection motivations and behaviors, while Lee et al. (2021) [28] found no significant correlation in this regard.

Consistent with the studies of Fathi et al. (2017) [38] and Hosseini et al. (2017) [29], in this study response costs had a weak negative correlation with protection motivation, while, in the study of Bashirian et al. (2020) [36], a positive significant correlation was reported between the intention of preventive behaviors and response costs; this is not consistent with the findings of the present study.

The results of this study indicate a significant positive correlation between perceived efficacy and the nurses' protective behavior. The findings of the study carried out by Mortada et al. (2021) [39] also showed that self-efficacy and response-efficacy were associated with the intention to participate in COVID-19 protective behaviors. In the study by Lee and Seomun (2021) [28], the nurses' coping appraisal which included response efficacy, self-efficacy had affected intentions to do security behaviors, but no direct effect was seen on the participants’ behavior, However, intention had moderating effect on the behavior. Our findings were consistent with the studies of Ismara et al. (2019) [35], Chen et al. (2020) [40], Fathi et al. (2017) [38], and Hosseini et al. (2017) [29].

Finally, in the present study, perceived threat showed a significant correlation with protective behaviors; however, Hosseini et al. (2017) [29] and Fathi et al. (2017) [38] reported a positive relationship between perceived the threats and protective behaviors. On the other hand, consistent with the findings of Ezati Rad et al. (2021) [37], in the present study, perceived fear showed no significant relationship with protective behaviors.

The results of this study showed that the protection motivation could explain 29% of the variance of adherence to safe injection guidelines in the hospital nursing staff. However, perceived rewards and perceived efficacy were the most powerful negative and positive predictors of motivation, respectively. Hosseini et al. (2017) [29] reported that PMT explained 32.6% of the variance of protective behaviors among laboratory personnel. In their study, perceived susceptibility was the most powerful predictor of protective behaviors. The studies on other protective behaviors revealed different predicting power of PMT; for example, this theory predicted 25% of breast self-examination behaviors in the Bashirian et al.’s (2019) study [41] and 64% of Covid-19 preventive behaviors in the study of Yazdanpanah et al. (2020) [42].

The structural equation model fit indices in this study represented an acceptable final model; Yazdanpanah et al. [42], Lee et al. [28], and Bashirian et al. [36] obtained similar results in their studies. Overall, given that nurses are a key member of the patient care team and their safety and health are important in advancing health goals [43], it is necessary to use appropriate theories for health and care planning.

## Limitations

Assessing compliance with needle stick guidelines through self-reporting and using online surveys were the most important limitations of this study that may lead to inaccurate results and information bias, in such a way that some respondents may not actually adhere to safe injection guidelines even though they reported they did so. Researchers may consider using mixed methods (e.g., observational methods and self-reports) to assess protective behaviors in future studies. Another limitation was unwillingness and weak cooperation of some members of the research community to participate in the study. Using cross-sectional design was another limitation of the study, and no inferences of causality can and should be made.

## Conclusion

The results suggest that the framework of the PMT was found useful for the prediction of NSIs preventive behaviors. Perceived efficacy, perceived effectiveness, and perceived rewards were the most important predictors of Adherence to Safe Injection Guidelines in the nursing staff. Thus, based on the results of the present study, it is suggested that development of educational materials and interventions for hospital nurses to comply with safe injection guidelines should be focused more on these variables.

### Supplementary Information

**Additional file 1.** Questionnaire.

## Data Availability

The datasets used and/or analyzed during the current study are available from the corresponding author on reasonable request.
