# Supplementary material for: Predictors of hospital nursing staff’s adherence to safe injection guidelines: application of the protection motivation theory in Fars province, Iran
Source: BMC Nurs. 2024 Jan 9;23:25. doi: 10.1186/s12912-023-01687-x (PMC10775610; doi:10.1186/s12912-023-01687-x)
Supplement: Supplementary file 1 — Additional file 1. Questionnaire. [file 12912_2023_1687_MOESM1_ESM.docx]

**Questionnaire**

**Please notice that the questionnaire just was translated to English and the standard process of translation re-translation has not done on it.**

How many times have you encountered each of the following during your career time?

not at all/ once/twice/three times/four and more times

- Sticking sharp objects into your hands or other parts of your body
- Spilling sick secretions on your face or hands
- Spilling the patient's blood on your face or hands

| Construct | Item | Strongly agree | Agree | No idea | disagree | Strongly disagree |
| --- | --- | --- | --- | --- | --- | --- |
| Perceived threats | Exposing the patient's blood and secretions during injections can lead to dangerous diseases. |  |  |  |  |  |
|  | Treatment of diseases caused by exposing with the patient's blood and secretions can be very difficult. |  |  |  |  |  |
|  | Treatment of diseases caused by exposing with the patient's blood and secretions can be impossible. |  |  |  |  |  |
|  | I am at risk of coming into contact with needle tips or patient secretions when providing services to patients. |  |  |  |  |  |
|  | I may contract blood-borne diseases from contact with needle tips or patient secretions while providing services to patients. |  |  |  |  |  |
|  | Considering that I am completely careful when giving injections, blood sampling, suturing, or other services to patients, there is no possibility for me to come into contact with the tip of the needle or the secretions of the patient. |  |  |  |  |  |
| Rewards | I work faster when I don't wear gloves when serving a patient |  |  |  |  |  |
|  | I see better when I don't wear protective glasses while providing patient care |  |  |  |  |  |
|  | It is easier to bend the tip of the needle and throw it in the trash than to throw it in a safety box. |  |  |  |  |  |
|  | My colleagues consider my non-use of safety equipment (gloves, glasses, etc.) when providing services to patients as a sign of my self-confidence and courage. |  |  |  |  |  |
|  | The hospital officials encourage me to save on the use of safety equipment (gloves, glasses, etc.). |  |  |  |  |  |
|  | When I don't waste patients' time due to safety issues during injections and dressings and other services, they are more satisfied. |  |  |  |  |  |
| Response costs | When the department is busy, observing safety precautions during injections and dressings causes time wasting and patient dissatisfaction. |  |  |  |  |  |
|  | Wearing a plastic apron reduces my mobility and the speed of service delivery. |  |  |  |  |  |
|  | The use of masks and glasses when giving injections causes anxiety and fear in patients. |  |  |  |  |  |
| Perceived efficacy | I can observe safety precautions in administering injections and dressings even when the department is busy. |  |  |  |  |  |
|  | I can maintain my concentration and do my work carefully while wearing glasses, mask and gloves.  I can avoid causing anxiety and worry in the patient by communicating properly while using glasses, mask and gloves. |  |  |  |  |  |
|  | Using glasses, masks and gloves during injections and dressings does not have much effect on protecting me against diseases. |  |  |  |  |  |
|  | I can prevent dangerous diseases by immediately reporting cases of contact with needle tips or patient secretions to the infection control supervisor or center manager. |  |  |  |  |  |
|  | Throwing the needles in a special container (Safety Box) has an effect on reducing my injury from the needle sticking into my hand. |  |  |  |  |  |
| Fear | When giving injections to patients, I worry about the needle going into my hand. |  |  |  |  |  |
|  | When doing dressings or stitches for patients, I worry about splashing the patient's blood and secretions on my face. |  |  |  |  |  |
|  | I get worried when I think about the injuries that may happen to me as a result of not observing the safety principles during injections |  |  |  |  |  |
| Protection Motivation | From now on, when you will do procedures such as injection, vein drawing, blood drawing, suturing or other such things for the patient, will you follow any of the following? | Not at all | Probably not | Possible | probably | definitely |
|  | Using a protective pad when breaking vials |  |  |  |  |  |
|  | Wearing gloves if you have cuts or wounds on your hands |  |  |  |  |  |
|  | Using glasses to protect the eyes from splashing blood and secretions of the patient |  |  |  |  |  |
|  | Using a mask to protect the mouth and face from splashing blood and secretions of the patient |  |  |  |  |  |
|  | Wearing a plastic apron to protect the body against contamination with blood or body secretions |  |  |  |  |  |
|  | Not putting a needle head cover on it |  |  |  |  |  |
|  | Avoid breaking or bending the needle head before disposing of it |  |  |  |  |  |
| Behavior | Currently, when you are doing procedures such as injection, vein drawing, blood drawing, suturing or other such things for the patient, do you follow any of the following? | Not at all | rarely | sometimes | always |  |
|  | Using a protective pad when breaking vials |  |  |  |  |  |
|  | Wearing gloves if you have cuts or wounds on your hands |  |  |  |  |  |
|  | Using glasses to protect the eyes from splashing blood and secretions of the patient |  |  |  |  |  |
|  | Using a mask to protect the mouth and face from splashing blood and secretions of the patient |  |  |  |  |  |
|  | Wearing a plastic apron to protect the body against contamination with blood or body secretions |  |  |  |  |  |
|  | Not putting a needle head cover on it |  |  |  |  |  |
|  | Avoid breaking or bending the needle head before disposing of it |  |  |  |  |  |
